# Balance and plantar cutaneous sensitivity functional assessment in community-dwelling elderly

**DOI:** 10.1590/S1808-86942010000200012

**Published:** 2015-10-19

**Authors:** Onivaldo Bretan, Rafael Martins Pinheiro, José Eduardo Corrente

**Affiliations:** Senior assistant professor, adjunct professor; Graduate student of General Basis of Surgery, physical therapist; Senior assistant professor, biostatistician

**Keywords:** elderly, posture, public health

## Abstract

Impaired balance is one of several factors that increase an elderly's susceptibility to falls. Balance assessment can be performed using postural tests and plantar cutaneous sensitivity tests.

**Aim:**

To assess balance disorders and loss of plantar cutaneous sensitivity in the elderly and look for association between these alterations.

**Materials and Methods:**

A descriptive cross-sectional study involving 45 elderly submitted to the Berg Balance Scale (BBS) and the plantar sensitivity test with nylon monofilament. We used chi-square and Fisher's exact tests, and ROC curves were created in order to study the sensitivity and specificity of BBS.

**Results:**

Two and 4 individuals showed balance and cutaneous sensation disorders, respectively. There was a significant association between the complaint of impaired balance and loss of skin sensitivity (p = 0.047), and there was a reasonable agreement (Kappa: 0.6457) between the BBS and the sensorial test. A significant association was also found among 6 of the 14 BBS tasks and the sensitivity test.

**Conclusion:**

Most of the elderly living independently in the community showed normal balance and plantar cutaneous sensation. When impaired, these functions appeared associated in a way that if the tests are performed together, the accuracy of the evaluation of the balance increases.

## INTRODUCTION

A fall is an event that occurs in a large percentage of the elderly; it causes fear of further falls, thus limiting daily activities. It is also a cause of significant morbidity and mortality.^1^, ^2^, ^3^ The Berg balance scale (BBS) and Tinetti's mobility score are functional clinical tests of balance used to assess the risk of falls in the elderly during their daily activities.^4^, ^5^, ^6^, ^7^ The BBS is more sensitive and specific, and is a reliable tool for measuring balance quantitatively in community-dwelling elderly persons.^7^, ^8^, ^9^, ^10^ It is an easy test to apply and makes it possible to directly evaluate the functional relevance of any change.^9^^,^^11^ The BBS has been validated for the Brazilian context.12 Scores and results vary widely in balance and risk of fall studies in community-dwelling elderly persons when using the BBS. This is due in part to biases incurred by authors when selecting subjects with different characteristics; individuals may have complaints of unbalance, systemic or neurologic diseases, use of drugs that act on the central nervous system, more elderly subjects, or specific exclusion criteria for the test.^1^^,^^5^^,^^7^^,^^8^^,^^13^

Studies have shown a correlation between altered plantar sensitivity and balance disorders among individuals with complaints of sensitivity, patients with neurologic or systemic disease - especially diabetes mellitus - in elderly subjects or not.^10^^,^^14^^,^^15^ Plantar skin sensitivity decreases significantly in healthy elderly persons; an association between loss of sensitivity and unbalance has been demonstrated in subjects with no complaints of insensitivity or unbalance.^16^, ^17^, ^18^ Studies on the association between balance and plantar sensitivity have generally applied sensitivity and balance laboratory tests with the Semmes-Weinstein nylons monofilaments,^19^, ^20^, ^21^ which are used mostly by clinicians and dermatologists. Monofilaments are sensitive and specific, and ideal as time-saving low-cost screening easily applicable in any setting.^18^^,^^19^ There are no published reports on a joint study of plantar skin sensitivity and balance in community-dwelling elderly persons that will have applied a balance functional test and nylon monofilaments, with no prior selection of subjects with complaints, diseases or factors leading to unbalance or altered plantar skin sensitivity.

The purpose of this study was to investigate the occurrence of unbalance and altered plantar sensitivity in community-dwelling elderly persons, by applying the BBS and nylon monofilaments. A second aim was to verify any association among test results and to note whether using both tests jointly could provide a more precise evaluation of balance.

## SERIES AND METHOD

A cross-sectional descriptive study of cases comprising 45 elderly subjects of both sexes was carried out to evaluate balance in community-dwelling subjects. These subjects were part of an elderly population clinical study that presented voluntarily upon request. The assessment was made randomly, according to the order of arrival. Exclusion criteria were: aged below 60 years, using any support for walking, orthopedic conditions of the lower limbs, diseases that affected gait, inability to understand instructions, major visual difficulty, a recent stroke, or transient ischemic attack. The institutional review board approved this study (number 2088/2006). Subjects signed a free informed consent form.

The BBS was applied as a functional balance evaluation test.^5^^,^^9^ It consists of 14 tasks similar to activities of daily life. Scores range from 0 (inability to carry out tasks without help) to 4 (ability to carry out tasks independently). The maximum score is 56 points; a 45 cutoff point was defined.^9^ This 45-point score separates subjects with a tendency to fall from those without.^7^

Plantar skin sensitivity was evaluated with a Semmes-Weinstein monofilament number 5.07; it generates a 10-g pressure when applied on the surface of the foot.^14^^,^^15^^,^^18^ The reasons for using this monofilament number have been previously described.^21^ The sensitivity of evaluation technique applied in this study was 97% and the specificity was 83% for lack of perception in four or more points to identify loss of protective sensitivity.^21^

The statistical analysis was aimed to check for any associations among variables for the plantar skin sensitivity test, question on unbalance and falls, and the BBS values; the chi-square test or Fischer's exact test were used, as appropriate. Reasons for use, the level and significance were 5% probability or the corresponding p-value. ROC curves were drawn based on BBS scores and the variables relating to questions on unbalance and falls within the last 12 months to increase the sensitivity of cut-off points. The number of subjects reporting unbalance and falls within the past 12 months was taken as the gold standard for calculations.

## RESULTS

There were slightly more females than males in this study; the mean age was 73 ± 8.05, as seen on Table 1. The mean BBS score was 48.89 ± 3.51, above the cut-off point adopted for separating subjects at risk of unbalance and falls. There were 4 (9%) elderly subjects with altered plantar sensitivity. There were 40 subjects with one or more systemic diseases, using corresponding medication. The most frequent diseases were arterial hypertension (58% of subjects), “heart diseases” (22%), “lung diseases” (15%), severe infection in the past (14%), diabetes (13%), and infarctions (7%). There were no reports of “strokes”. Difficulties with vision (71%), falls in the past (42%), and unbalance (31%) were other reported conditions among the elderly subjects. Systemic medication was used by 75% of subjects, and current or previous depression was reported by 18% of subjects.

**Table 1:** Clinical and social & demographic data (n=45).

Male	21 (47%)	
Female	24 (53%)	
Age (mean ± standard deviation)	73 ± 8,05	
Cut-off point in the BBS (mean ± standard deviation)	48,89 ± 3,51	
Subjects with one or more systemic diseases	40	
Subjects with altered plantar skin sensitivity	4 (9%)	
	YES	%
Do you have high blood pressure?	26	58
Do you have diabetes?	6	13
Do you have high blood cholesterol?	13	23
Do you have heart disease?	10	22
Have you had an infarction?	3	7
Have you had a stroke?	0	0
Do you have kidney disease?	3	7
Do you use medication?	34	75
Are you or have you been depressed?	8	18
Do you have lung disease?	7	15
Do you have problems with vision?	32	71
Have you had any severe infection?	2	14
Have you had vertigo?	17	38
Do you have unbalance?	14	31
Have you fallen at any time in the past?	19	42

Association studies of subjects complaining of unbalance or falls in the previous year and the plantar sensitivity test have shown statistical significance in relation to “unbalance” (p = 0.047). Among 45 subjects, 14 reported unbalance, of which three had altered plantar sensitivity. Table 2 shows the distribution of subjects according to the BBS cutoff point chosen initially and skin sensitivity. The cohort score (45) revealed that 43 subjects scored ≥ 45, and 2 subjects scored < 45 - about 5% of the elderly subjects. The kappa coefficient showed reasonable agreement between the BBS and the plantar sensitivity test.

**Table 2:** Distribution of subjects according to the BBS scores and the plantar skin sensitivity test (cut-off score: 45).

BBS	Plantar sensitivity		Total
	Normal	Altered	
≥ 45	41	2	43
< 45	0	2	2
Total	41	4	45

Kappa coefficient = 0.6457 (95% confidence interval: 0.1968-1.000)

The ROC curve took into account the BBS scores and the number of subjects reporting unbalance, and showed that 48 was a more relevant score for 0.677 specificity and 0.714 sensitivity. There were 37 subjects with scores ≥ 48 and 8 with scores < 48. The kappa coefficient, however, showed low agreement between tests. The ROC curve on the BBS scores and the number of subjects reporting falls showed that 49 was the most relevant scores for a 0.677 specificity and a 0.714 sensitivity. There were 37 subjects with scores ≥ 49 and 8 with scores < 49. The kappa coefficient showed poor agreement among the tests (Table 3). An overview showed that plantar sensitivity was altered in subjects with scores above or below the cutoff points 45, 48 and 49.

**Table 3:** Distribution of subjects reporting unbalance and falls according to the BBS score and the plantar skin sensitivity test.

UNBALANCE			
BBS	Plantar sensitivity		Total
Cut-off score: 48	Normal	Altered	
≥ 48	35	2	37
< 48	6	2	8
Total	41	4	45
**FALL**			
BBS	Plantar sensitivity		Total
Cut-off score: 49	Normal	Altered	
≥ 49	35	2	37
< 49	6	2	8
Total	41	4	45

Kappa coefficient = 0.2437 (95% confidence interval: 0-0.6058)

Kappa coefficient = 0.2437 (95% confidence interval: 0-0.6058)

Association studies between BBS and plantar sensitivity were done by confronting the scores of each one of the 14 tasks with the number of points in the sensitivity test. Table 4 shows that there were statistically significant results in 6 of 14 tasks (BBS); this means that a positive association with the plantar sensitivity test was found in 40% of tasks.

**Table 4:** Association between the BBS and the plantar sensitivity test (n = 45).

BBS	N° subjects			Total	p-value	BBS	Score	N° subjects		Total	p-value
		Normal Sensitivity	Altered Sensitivity					Normal Sensitivity	Altered Sensitivity		
Sitting to standing	0	0	0	0	0,2355		0	0	0	0	0,7943
	1	0	0	0		Recline forward with arms extended	1	0	0	0	
	2	0	0	0			2	1	0	1	
	3	3	1	4			3	14	2	16	
	4	38	3	41			4	26	2	28	
	total	41	4	45			total	41	4	45	
	0	0	0	0	(-)		0	0	1	1	<,0001
Standing with no support	1	0	0	0		Pick up object on floor	1	1	0	1	
	2	0	0	0			2	0	1	1	
	3	0	0	0			3	2	1	3	
	4	41	4	45			4	38	1	39	
	total	41	4	45			total	41	4	45	
Sitting with no back support	0	0	0	0	(-)	Standing, turn and look backwards over right or left shoulder	0	1	0	1	0,4072
	1	0	0	0			1	0	0	0	
	2	0	0	0			2	2	1	3	
	3	0	0	0			3	6	0	6	
	4	41	4	45			4	32	3	35	
	total	41	4	45			total	41	4	45	
	0	0	0	0	0,0137		0	0	0	0	0,0178
	1	0	0	0			1	1	0	1	
	2	0	0	0		Turn 360 degrees	2	2	2	4	
	3	8	3	11			3	6	1	7	
	4	33	1	34			4	32	1	33	
	total	41	4	45			total	41	4	45	
	0	0	0	0	0,0095		0	0	1	1	0,0016
Transference	1	0	0	0			1	0	0	0	
	2	0	0	0		Placing feet alternatively on a bench	2	0	0	0	
	3	3	2	5			3	9	2	11	
	4	38	2	40			4	32	1	33	
	total	41	4	45			total	41	4	45	
Standing with eyes closed	0	0	0	0	0,5755		0	1	1	2	0,2068
	1	0	0	0		Standing with one foot facing the other	1	3	0	3	
	2	0	0	0			2	1	0	1	
	3	3	0	3			3	12	0	12	
	4	38	4	42			4	24	3	27	
	total	41	4	45			total	41	4	45	
Standing with feet next to each other	0	0	0	0	0,0137		0	0	0	0	0,7075
	1	1	0	1			1	5	1	6	
	2	0	1	1		Standing supported on one leg	2	9	0	9	
	3	2	0	2			3	8	1	9	
	4	38	3	41			4	19	2	21	
	total	41	4	45			total	41	4	45	

## DISCUSSION

The number of elderly subjects reporting systemic conditions, falls, unbalance, vertigo, problems with vision, and use of systemic medication was high in our clinical assessment, and was close to other published results.^1^^,^^8^^,^^15^^,^^22^ Each one of these disorders is an independent predictor of risk of falls.^1^^,^^15^^,^^18^^,^^19^ In this study, the number of subjects with BBS scores below 45 was small; the mean was about 49, close to the scores of control group elderly subjects in studies on the risk of falls.^7^^,^^8^^,^^11^

Plantar skin sensitivity studies in several systemic and neurologic diseases show a wide range of values in the different evaluation methods used by the authors. Such values were at least 100% higher compared to control group results.^14^^,^^15^ Diabetes mellitus is the most frequent cause of loss of sensitivity due to peripheral neuropathy; about 50% of diabetics aged over 60 years have loss of the protective plantar sensitivity due to neuropathy.^14^^,^^15^^,^^23^ It has also been demonstrated that loss of plantar sensitivity in apparently healthy elderly subjects with no complaints is due to subclinical peripheral neuropathy.^22^^,^^23^ Baseline clinical data available in the present study only allow us to suggest diabetes (found in 13% of subjects) on the one hand, and the low mean age, on the other, as the most important factors; these are possibly accountable for the low number of sensory changes found in the sample.

Calculation of BBS specificity and sensitivity relative to the percentage of subjects complaining of unbalance and falls revealed cut-off points of 48 and 49 respectively. However, agreement between both tests was low, although good agreement was attained with the previously defined cut-off point, which was considered more appropriate for this study. Poor agreement in the complaint unbalance in a way conflicts with the significant association found between unbalance and loss of plantar sensitivity; it diverges from a study showing that altered BBS scores and complaints of unbalance are predictors of falls.^8^ In this study, not only was plantar skin sensitivity associated with a complaint of unbalance, but there was also an association between BBS and plantar sensitivity results. This study revealed a previously unpublished close relation between balance and sensitivity after confronting the scores of each one of 14 BBS tasks with plantar sensitivity tests.

A single pilot study came close to our assessment; it applied the Tinetti functional mobility test and monofilaments in 12 diabetics of different ages in urban and rural-dwelling communities.^15^ The authors found altered balance and decreased plantar sensitivity respectively in 40% and 100% of 7 urban dwellers and in 18% and 67% of 5 rural dwellers. The significant difference and the small number of subjects in both studies suggest a need for further investigation with larger samples to confirm these results. Studies on balance in elderly subjects that have focused on specific health issues (physical, mental and others) have generated useful data. Non-directed studies, however, are also important to learn about unbalance in the elderly as a whole to support public policies for preventing falls and providing rehabilitation of balance measures.

This study showed that the tests applied above are good assessment tools. Routine and periodic observation of balance and sensitivity is desirable, since falls become more frequent with age; about 30% of individuals aged from entre 65 and 74 years and 40% of individuals aged 75 years or more are affected.^22^^,^^24^^,^^25^ Clinical healthcare professionals should be attentive to the risk of falls in their patients and refer them to balance specialists.

A limitation of this study - aside from the small sample size - was the method used to make contact with the subjects. It is possible that only younger and healthier individuals, with controlled diseases and independent lives, may have presented.^26^ More encompassing studies are required to generalize these results. A second limitation of this study was the impossibility of carrying out a vestibular assessment, which would have been important not only due to a significant number of subjects responding positively to the question on vertigo, but mainly because this complaint is common in the elderly.^27^ A correlation between otoneurology assessment data and the present results could further our understanding of the events observed. However, operational difficulties, including the scheduled time for completing the population study, made further investigation impossible. It should be emphasized that studies on unbalance that aim to investigate one or more of its multiple causes should take into account the significant incidence of vestibular diseases among the elderly, and always count on the support of an otoneurologist, as in the present study.

## CONCLUSION

Elderly community-dwelling subjects with independent lives for the most part have normal plantar skin sensitivity. Subjects with postural disorders in which these functions are altered may be assessed more accurately by associating functional balance tests with plantar sensitivity tests.
